# Multisectoral Approach to Support Use of Insecticide-Treated Net for Malaria Prevention Among Mobile and Migrant Populations in Myanmar: A Systematic Review

**DOI:** 10.1093/infdis/jiaa335

**Published:** 2020-10-29

**Authors:** Cho Naing, Maxine A Whittaker, Marcel Tanner

**Affiliations:** 1 International Medical University, Kuala Lumpur, Malaysia; 2 Division of Tropical Health and Medicine, James Cook University, Townsville, Queensland, Australia; 3 Swiss Tropical and Public Health Institute, Basel, Switzerland

**Keywords:** malaria, prevention, bed nets, multisectoral approach, artemisinin resistance containment zones, Myanmar

## Abstract

**Background:**

Myanmar is a premalaria elimination country with artemisinin-resistant malaria. A strategy for transmission control is focused on vulnerable groups such as mobile and migrant populations (MMPs), and includes improving access to insecticide-treated bed nets in the Myanmar artemisinin resistance containment (MARC) zones using multisectoral approaches (MSA).

**Methods:**

This narrative systematic review addressed MSAs targeted to MMPs in Myanmar for malaria prevention. We searched relevant studies in electronic databases and present the narrative findings in 4 domains: stakeholder groups, net coverage and utilization, social determinates, and facilitators/barriers.

**Results:**

Nine studies were included. The review identified stakeholders involved in intersectoral collaboration. Net ownership was higher than utilization rates in the MARC zones and rates remained below the WHO recommended target of 100%. There was inadequate description of roles and responsibilities for implementation and on channels of communication within the partnerships and with the Government.

**Conclusions:**

Findings show that interventions to distribute treated bed nets were supported by the multiple stakeholders. Due to the design of the primary studies, analysis of the added value of intersectoral collaboration was limited. More attention must be paid to designing studies to document and evaluate the contributions and outcomes of intersectoral collaboration.

The World Health Organization (WHO) 2030 Agenda for Sustainable Development goal 3 is committed to “ensure healthy lives and promote well-being for all, at all ages,” while goal 10 is “to reduce inequity within and among countries” [1, 2]. One of the groups that experiences inequality in attaining general well-being is the mobile and migrant population (MMP) and there have been calls by international agencies like the WHO and International Organization for Migration (IOM) to focus specifically on their needs [2, 3]. In Myanmar, the health authorities and WHO have noted that in addition to people who live in the forest and/or reside in forest fringe villages, “MMPs, who are often induced by economic opportunities such as logging or mining in forested areas or road or dam construction and maintenance, are considered the major risk group for malaria transmission” [3]. MMPs, in the context of malaria in the Greater Mekong Subregion (GMS) are “characterised predominantly by international migrants, seasonal and permanent migrant workers, as well as internal migrants, displaced persons and refugees. Internally displaced persons and refugees are found in these countries as are mobile and migrant populations moving within and across countries of the GMS.” These MMPs have limited or no access to health resources such as formal health facilities due to many factors such as their illegal migration status, language and cultural barriers, antimigrant sentiments, and lack of migrant-inclusive health policies [4].

Myanmar, which is located in Southeast Asia, is in the preelimination stage of malaria. A major strategy of malaria transmission control in Myanmar includes a focus on the vulnerable groups such as MMPs. In 2014, 9.4 million people were internal migrants (defined as intertownship movement of more than 6 months), which was equivalent to 20% of the total population [5].

As occurs elsewhere among the preelimination countries, malaria transmission often becomes increasingly focused in high-risk groups, which in Myanmar include MMPs, who are concentrated in the regions with multidrug-resistant *Plasmodium falciparum* [6, 7]. The Myanmar artemisinin resistance containment (MARC) strategy, which is endorsed by the WHO, aimed to implement various efforts to contain the spread of artemisinin resistance in Myanmar with emphasis on MMPs [6]. Along this line, the WHO global technical strategy [7, 8] has recommended full coverage of treated bed nets (here referred to as treated nets) in the form of insecticide-treated nets (ITNs) and long-lasting insecticidal nets (LLINs) for malaria prevention [9]. Although, more than 1 method of vector control exists, the WHO has recommended attaining high coverage of a single method before a second form is deployed [10]. A Cochrane systematic review documented that ITNs could reduce the prevalence of *P. falciparum* malaria by 17% compared to the absence of nets [11].

In practice, the opportunities for distribution of nets to MMPs, who the health service find hard to reach, needs to channel efforts through multisectoral approaches (MSA) across health and nonhealth sectors, such as the IOM [4, 9, 12]. Collaborative partnerships (people and organizations from multiple sectors working together towards a common purpose) are a prominent strategy for community health improvement [13]. Although the interventions related to treated net distribution/utilization targeted to MMPs through MSA have increased over time, significant gaps exist in reported evidence. The use of multisectoral interventions in public health raises the issue of what constitutes evidence in the other sectors, and which part of the evidence plays a significant role in their decision-making processes [13]. This review focused on 3 central questions: (1) what specific sectors are involved in an intervention of distribution of treated nets among MMPs in the MARC zones?; (2) what value do collaborative partnerships bring to implementation of these interventions for MMPs?; and (3) what factors (barriers or facilitators) contributed to the success of the partnership and the intervention outcomes? Therefore, the objectives were to (1) summarize the participation of stakeholders to increase the coverage and use of the treated bed nets targeted to MMPs for malaria prevention, (2) summarize barriers/facilitators encountered in MSA targeted to MMPs for malaria prevention, and (3) identify the added value of a MSA.

## METHODS

This narrative systematic review addressed MSA targeting MMPs in Myanmar for malaria prevention.

### Search Strategy

We searched relevant studies in electronic databases (MEDLINE, Embase, Scopus, Global Health, ERIC, Social Science Citation Index, and Cochrane Library), freely available internet search engine (Google scholar), and relevant websites (including Myanmar Ministry of Health [MOH], WHO Myanmar, WHO South-East Asia Regional Office, and United Nations High Commissioner for Refugees websites). We also contacted the researcher in whose article the study was reported if there was incomplete data for the review’s analysis to request the provision of the missing information and for clarification of data sources and quality. Keywords were developed through meetings between the research team and a librarian who was involved in a larger study on MSA targeted to MMPs [14]. The search strategy used for PubMed is provided in Supplementary Table 1. The search strategy used in PubMed was modified for use in other databases. Our search was limited to studies reported in English language published between January 2000 and July 2019.

### Inclusion Criteria

Eligible studies were selected based on the PICO format, as follows.

Study population (P): MMPs in the MARC zones. MMPs for this particular study were operationally defined as those who were internal migrants (eg, seasonal laborers, forest workers), internally displaced persons (individuals who have been forced to leave their homes or places of habitual residence, but who have not crossed an international border [15]), nonpermanent migrants (nonlasting residence at the location [16]), and cross-border migrants. We excluded personnel employed in the armed forces and permanent migrants from MMPs due to the particular characteristics of their work and/or situation.
Study intervention/exposure (I): Treated nets in the form of ITN or LLINs for malaria prevention.
Study comparator (C): All quantitative and mixed-methods designs were considered.
Study outcome (O): The study reported data on the process of MSA or at least 1 outcome attributed to intervention. The outcomes considered were the domains on stakeholder groups, net coverage and utilization, social determinants, and facilitators/barriers that were encountered in MSA. Detailed descriptions are presented in Supplementary Table 2.
Study design: Observational studies that reported at least 1 outcome.

Studies were excluded if they: (1) were not reported in English, (2) did not describe the MSA, and (3) were not targeted to the MMPs in the MARC zone.

### Data Extraction

One investigator (C.N.) screened the titles and abstracts and selected the relevant full-text articles, following the inclusion criteria. Two investigators (C.N. and M.A.W.) independently extracted data from each study, using a piloted data extraction form. Information collected included: first author, publication year, study region, study design, sample size, intervention, sectors involved, main findings, and type of support. Any discrepancy between the 2 investigators was resolved by consensus. We did not evaluate the quality of studies in this narrative review of qualitative synthesis.

### Data Syntheses

For each study, to understand the contextual factors attributed to the treated net intervention among the MMPs, we narratively presented the findings in 4 domains (stakeholder groups, net coverage and utilization, social determinates, and facilitators/ barriers) when these were reported qualitatively in the studies. Due to the variability of study designs among the studies, a conventional aggregated meta-analysis was not possible.

## RESULTS

**Figure 1.**
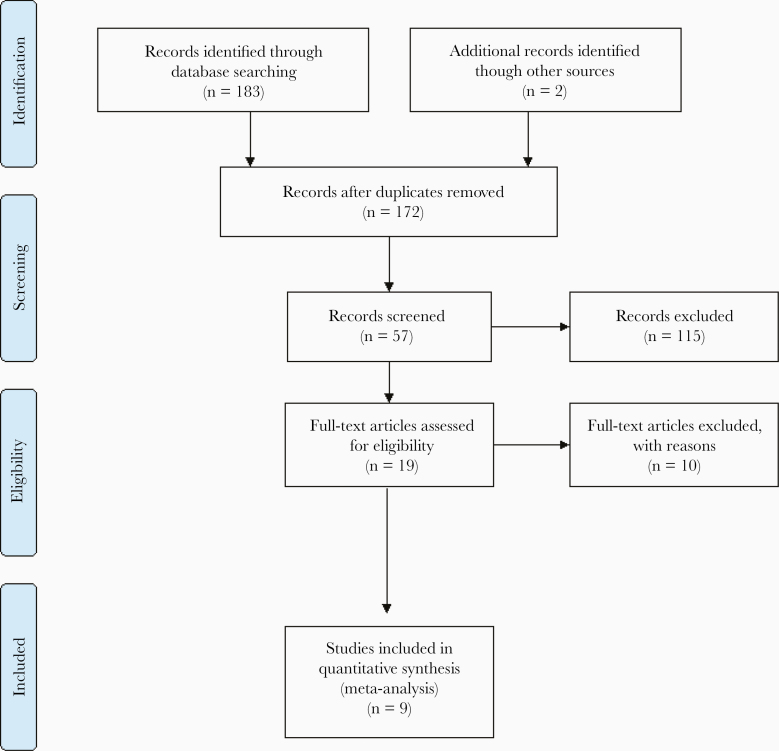
PRISMA flow chart of the study selection process.

The study selection process in a PRISMA flowchart is illustrated in Figure 1. Nineteen full-text articles were assessed for potential eligibility. Of these, 9 observational studies were included, consisting of data for 9203 households and 18 601 individuals belonging to MMPs in the MARC zones across Myanmar [17–25]. The 10 excluded studies and the main reasons for exclusion are provided in Supplementary Table 3.

All included studies had a cross-sectional design and used a combination of qualitative and social research methodologies, such as interviews and surveys with MMPs.

### Location of Studies

**Figure 2.**
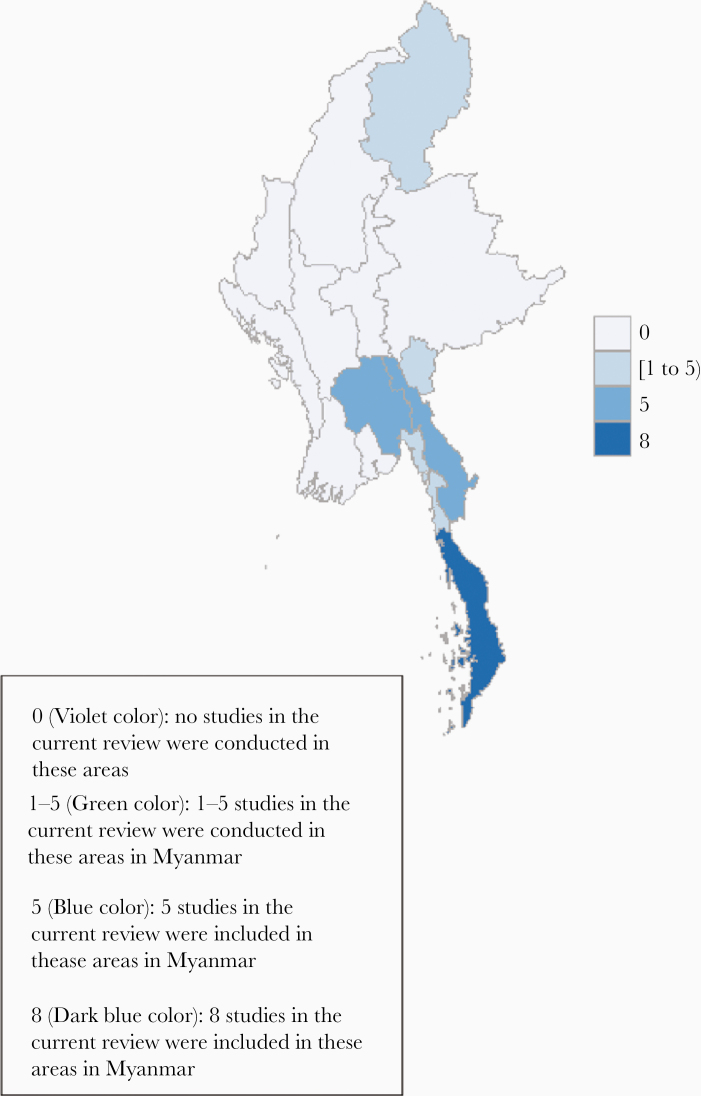
Distribution of included studies conducted in Myanmar.

All the included studies were undertaken among MMPs in the MARC zones in various administrative regions of Myanmar (Figure 2). Of the 9 studies, 6 were undertaken in more than 1 MARC zone [17, 18, 20, 21, 23, 25], while a single study was done in a MARC zone of Bago region [22] and 2 studies in Tanintharyi region [19, 23]. The publication years ranged from 2014 to 2019.

### Stakeholder Groups in Multisectoral Approaches

The included studies reported that multisectoral collaboration among various stakeholder groups (eg, government, private sector, volunteers, UN agencies, and aid agencies) and sectors (eg, health, industries, nongovernmental organizations [NGOs], community, and philanthropy) was utilized to jointly work on the interventions (treated nets) targeted to the MMPs with the shared aim of increased bed net use and coverage (Table 1). Almost all studies were funded by the government sector pertinent to the Myanmar MOH and the WHO. There was a common shortage of information on the role of each partner and the mechanisms of their involvement. The description of the specific NGO was also missing in many of these studies. Only the study by Wai et al [19] provided the particulars, namely that a local NGO (Myanmar Medical Associations) and 2 international NGOs (Population Service Internal and World Vision Myanmar) were involved in these activities targeted towards MMPs.

**Table 1.** Description of Sectors Involved in Treated Nest Intervention Targeted to Mobile and Migrant Population

Description	Sector Entity	MARC, 2012 [17]	Nyunt, 2014 [18]	Wai, 2014 [19]	Hlaing, 2015 [20]	Nyunt, 2015 [21]	Phyo Than, 2017 [22]	Soe, 2017 [23]	Maung, 2018 [24]	Linn, 2019 [25]
MOH^a,b,c^	Government	✓	✓	✓	✓	✓	✓	✓	✓	✓
WHO or WHO/TDR^b,c^	UN	✓	✓	✓	✓	…	✓	…	…	✓
GFATM^c^	International funding agency	✓	…	✓	…	…	…	…	✓	…
3DF^c^	International funding agency	✓	…	✓	✓	…	…	…	…	…
IOM^c^	UN	✓	…	…	…	…	…	…	…	…
INGO^a,c^	Nonprofit organization	…	…	✓ PSI, WV	…	…	…	…	…	✓ MSF; UNION
NGO^a,c^	Nonprofit organization	…	✓	✓ MMA	✓	✓	…	…	✓	✓
SORT-IT^b,c^	WHO	…	…	…	…	…	✓	…	…	✓
Community volunteers^a^	Nonprofit	…	…	✓	…	…	…	✓	…	…
Private vendors^a^	Private sector	…	…	…	…	…	…	…	…	✓
Burnet Institute, Australia^b,c^	Research institute, international NGO part of its work	…	…	…	…	…	…	…	…	✓
MIMU^d^	Nonprofit	…	…	✓	…	…	…	…	…	…
Others (gift)^a^	Philanthropy	…	…	…	…	…	…	…	…	✓

Abbreviations: INGO, international nongovernmental organization; MIMU, Myanmar Information Management Unit; MOH, Ministry of Health and Sports (Department of Health and Department of Medical Research for this particular study); MMA, Myanmar Medical Association; NGO, nongovernmental organization; PSI, Population Service Internal; UNION, International Union Against Tuberculosis and Lung Disease; WV, World Vision Myanmar.

^a^Supply and/or services.

^b^Research.

^c^Funding.

^d^Specific technology.

Three studies in this review identified merely the stakeholders involved used in the MSA for malaria prevention targeted to MMPs in these MARC zones [21, 24, 25]. One study reported that 63.3% of resources (distribution of treated nets) were from the MOH, while 31.3% were from the NGOs [24]. Another study showed that 60% and 5.8% of treated nets were supplied freely from the MOH and NGOs, respectively, and a small portion were purchased from private markets (0.5%) as well as gifts from unidentified sources (1.8%) [25]. The study by Nyunt et al reported that 26% and 10% treated nets were distributed by health staff and NGOs, respectively [21]. These studies did not identify the particular NGOs but described the contexts indicating multisectoral involvement.

### Net Ownership and Usage Levels

Four studies reported on both utilization and ownership [19–22, 25]. These studies showed that utilization of treated nets was lower than the ownership (Table 1). For example, the study by Linn et al [25] reported that the utilization of treated nets (36%; 95% confidence interval [CI], 15%–57%) was lower than the ownership (74%; 95% CI, 49%–93%).

Similarly, Phyo Than et al [22] reported a substantial rate of ownership (95%) with a very low rate of adequate utilization (ie, 1 bed net per 2 persons) for seasonal migrants (9%). Another study reported that 75.1% of households had at least 1 ITN but only 58.5 % had an adequate number of ITNs [21]. On the other hand, 1 study documented a low level of ownership (46.6%) as well as utilization rate (38.8%) [20].

Factors that may contribute to this major difference between owning a net and using it may include convenience of use (if sleeping outside the household), acceptability of use, perceived low levels of mosquitoes/risk, and awareness of the need for regular use. Specific to this, 1 study showed that a higher knowledge on causes of malaria was associated with higher utilization of the bed net while sleeping (odds ratio [OR], 4.04) [21]

### Treated Nets and Social Determinants

Only 1 study reported data on malaria and its associated factors such as “not sleeping under net” (OR, 2.02; 95% CI, 1.15–3.52) or the use of “torn bed net/net with large holes” (OR, 2.2; 95% CI, 1.21–3.33), which created around a 2-fold increase in risk of getting malaria among MMPs in the MARC zones [23]. Another study reported willingness to pay for the ITNs was 65.8% [23]. The study by Wai et al reported that the willingness to pay was 86.5% and the ability to pay was 60% [19]. This finding reflects an equity concern about the affordability of treated nets among MMPs.

### Barriers Encountered

The common barriers encountered were in the coverage and the use of treated nets, despite the support provided through MSA. One barrier was incomplete knowledge of the proper use and care of the nets. One study reported that 31.3% of the supported nets had holes and 38.9% of treated nets were in need of retreatment [24]. Another study documented that insufficient number of nets was a barrier: “I am alone and got one net, my son’s family has 7 members but he got only one net which was not sufficient for them” [25]. The underutilization of treated nets might be related to inadequate knowledge about malaria transmission and therefore prevention of transmission [21] and issues that affected acceptability of nets such as smell and reported skin irritation from use of the net.

Accessibility was another concern, as described. Physical accessibility is an issue as typified by 1 study’s description, “Reaching the place is very difficult, sometimes only by walking, need to carry nets and other things on the back. Even motorcycle won’t go that far” [25]. Women who prefer to access services from female providers was also an issue in hard to reach locations, as 1 study noted, “For riverine route, we do not want women as volunteers as the route is dangerous” [25]. The timing of the services such as net distribution could also prove a barrier, “I sleep under ordinary bed nets but not ITNs because I was not here when ITNs were distributed” [25]. Moreover, the services provided needed to be aligned with appropriate targeting of activities like behavior change communication to address special needs for groups such as people who, due to work or social activities, are out of the house during the night [25].

To address these barriers to access and utilization of nets, better planned programmatic support through collaborative initiatives among sectors is required. Wai and associates noted the plea of one community’s members, “We need collaborative work between health department and administrators to inform and motivate the regular use of LLIN,” which typifies this need [19].

## DISCUSSION

The current review provides some evidence on multisectoral collaborations that were involved in treated bed net intervention for the purpose of malaria prevention targeting MMPs in the MARC zones. The review found that there is a broad range of many stakeholders (ie, local and international agencies, NGOs, private sectors, employers of the migrant workers), who have supported treated net interventions for malaria control/elimination targeting high-risk populations. Although there were limited details in the included studies, the reported and described intersectoral collaboration seems to have made a contribution to some of the intermediate outcomes towards elimination, such as distribution and usage of treated nets, as well as accessibility of malaria diagnosis and treatment services (not explored in this review). However, just because there are authors of these studies from multiple agencies it does not necessarily reflect that their interactions when delivering the interventions were collaborative and equal partnerships, in the true intersectoral collaboration sense [26].

The selected studies were from the regions where the majority of the containment project interventions, such as LLIN distribution, have been implemented. The implementation covered MMPs but could not achieve the planned universal coverage level. Several reasons may explain this gap, including inadequate knowledge about the protective effects of treated nets, limited access to the treated nets, or ineffective scheduling of services challenging the access to and by the individuals from MMPs. Consequently, equity issues remain a concern, despite the efforts targeted to the MMPs through these multisectoral interventions. Historically, the link between malaria transmission and human population movement has been acknowledged [27, 28]. The failure of malaria eradication campaigns in the 1950s and 1960s was attributed to the failure to consider human population movement as an important determinant of transmission [29]. In the context of an increasing global interest in malaria elimination, MMPs communities will add an additional dimension to the role of formal health systems in improving population health.

### Implications

The findings from this review have practical implications for collaborative partnership in the context of malaria prevention. MSA is a powerful tool for improving effectiveness and efficiency of public health programs [18], such as treated net utilization in this case. Universal coverage for malaria vector control is defined as universal access to and use of appropriate interventions by populations at risk of malaria [30]. Along this line, universal coverage for ITNs is measured as the proportion of households with at least 1 ITN for every 2 people, meaning that if 1 net is given for every 2 people in a household, all members have a chance to use an ITN [31, 32].

As the coverage as well as utilization of treated nets are still below the universal coverage level of effective net ownership, more work is needed to meet the targeted level of net utilization and MSA can help achieve this goal. Importantly for smooth implementations of MSA, clear cut roles and responsibilities at various levels and with stakeholders need to be defined, with the individual and combined performance being measured against the defined tasks at each level [13].

The key strengths of this study include the robust systematic methodological approach used and meta-analysis of available data to provide the first evidence that MSA has been implemented in treated nets intervention among MMPs exclusively in the MARC zones. An earlier review highlighted that human population movement links several localities, which may have different malaria transmission levels and risks [33]. The current study addressed a specific population in a specific region, the MARC zones, giving insights for policy makers in better formulation of strategies in malaria elimination.

Much of the information retrieved from the studies involved in the review are descriptive, covering short periods with lack of follow-up for rigorous evaluation of the intervention. Also, we could not find studies reporting systematic measurement of individual sectors among the multisectoral partnerships. Another published systematic review on intersectoral action for health equity concurs with this finding, reporting that that the studies included were descriptive and the programs were not rigorously evaluated [34].

The surveys included in the present review were not designed primarily to address specifically MMPs. Thus, more specialized survey methodologies may be needed [17]. Due to the variations in study aims, sample size, study sites, aims of stakeholders involved, and methodological approaches in the studies, the current analyses are inevitably limited to a simplification and summary, rather than a robust assessment. Further, as there were often other interventions launched in the study sites, there may be confounding effects. As an example, a systematic review on nonhealth-targeted policies showed some impact on migrants’ health [35]. There is also the possibility that non-English language studies, nonpublished studies or reports, and books or websites that may shed light on this topic were overlooked.

Nevertheless, many of these findings may be applicable to the general MMPs, regardless of the type of health intervention. Despite methodological limitations, the analyses of multiple studies in the MARC zones may help us better understand the challenges and impact of the MSA interventions on the use of treated nets by MMPs.

### The Road Ahead

In designing future studies some limitations that we identified in the literature should be considered. More robust research, using standardized methodologies, reporting, and follow-up assessments are needed to accurately assess the health status of MMPs in the malaria prevention context and explore options for health policy direction. Moreover, examining health outcomes over a longer period and the subsequent impact on MMPs across all studies are required. In addition, a clear description of the required roles of stakeholders and the channels of communication within the sectors to ensure the success of the interventions are clearly outlined.

## CONCLUSIONS

The findings show that interventions targeted to MMPs to distribute ITNs were supported by the multiple stakeholders. Due to the nature of the study design of the primary studies in the present review, the findings are inevitably a simplification, summary, and collection of information. For a better understanding of the added value of intersectoral collaboration more attention must be paid to designing studies to document and evaluate the contributions and outcomes of intersectoral collaboration.

## Supplementary Data

Supplementary materials are available at *The Journal of Infectious Diseases* online. Consisting of data provided by the authors to benefit the reader, the posted materials are not copyedited and are the sole responsibility of the authors, so questions or comments should be addressed to the corresponding author.

jiaa335_suppl_Supplementary_Table_1

jiaa335_suppl_Supplementary_Table_2

jiaa335_suppl_Supplementary_Table_3

## Notes

***Acknowledgments.*** We thank the anonymous reviewers and the editors for the comments and inputs provided. We are grateful to Dr Florence Fouque and Dr Qingxia Zhong (UNICEF/UNDP/World Bank/WHO Special Programme for Research and Training in Tropical Diseases) for their continuous support throughout the study process.

***Disclaimer***. The funder had no role in study design, data collection, data analysis, data interpretation, or writing of the report.

***Financial support.*** This work was supported by UNICEF/UNDP/World Bank/WHO Special Programme for Research and Training in Tropical Diseases (TDR) (grant number 2017/721367-0).

***Supplement sponsorship.*** This Supplement is sponsored by TDR: the Special Programme for Research and Training in Tropical Diseases co-sponsored by the United Nations Children’s Fund (UNICEF), the United Nations Development Programme (UNDP), the World Bank and the World Health Organization (WHO).

***Potential conflicts of interest.*** All authors: No reported conflicts of interest. All authors have submitted the ICMJE Form for Disclosure of Potential Conflicts of Interest. Conflicts that the editors consider relevant to the content of the manuscript have been disclosed.

Presented in part: First Malaria World Congress, 1–5 July 2018, Melbourne, Australia.
