# Supplementary material for: Multisectoral Approach to Support Use of Insecticide-Treated Net for Malaria Prevention Among Mobile and Migrant Populations in Myanmar: A Systematic Review
Source: J Infect Dis. 2020 Oct 29;222(Suppl 8):S717–25. doi: 10.1093/infdis/jiaa335 (PMC7594345; doi:10.1093/infdis/jiaa335)
Supplement: jiaa335_suppl_Supplementary_Table_1 [file jiaa335_suppl_supplementary_table_1.doc]

**Supplementary Table 1**. PUBMED Search string

| (((("malaria"[MeSH Terms] OR "malaria"[All Fields]) AND ("transients and migrants"[MeSH Terms] OR ("transients"[All Fields] AND "migrants"[All Fields]) OR "transients and migrants"[All Fields] OR "migrants"[All Fields])) OR mobile[All Fields]) AND (("beds"[MeSH Terms] OR "beds"[All Fields] OR "bed"[All Fields]) AND net[All Fields])) AND ("myanmar"[MeSH Terms] OR "myanmar"[All Fields]) |
| --- |
