# Supplementary material for: Multisectoral Approach to Support Use of Insecticide-Treated Net for Malaria Prevention Among Mobile and Migrant Populations in Myanmar: A Systematic Review
Source: J Infect Dis. 2020 Oct 29;222(Suppl 8):S717–25. doi: 10.1093/infdis/jiaa335 (PMC7594345; doi:10.1093/infdis/jiaa335)
Supplement: jiaa335_suppl_Supplementary_Table_2 [file jiaa335_suppl_supplementary_table_2.doc]

Supplementary Table 2. Inclusion criteria

| Study outcome | At least one outcome such as knowledge acquisition, coverage, uptake, effectiveness, factors influencing (barriers or facilitators) the implementation these mechanisms either quantitative or qualitative measure related to ITNs for malaria prevention targeted to the MMPs. Unit of analysis was either household member or household. Households represents both formal as well as in-formal structure [25]. Net-owning and net-utilization are as defined in the primary studies. We preferred temporary work setting that covered without any structure and moving around for better economic opportunities [23]. |
| --- | --- |
| Study comparator/designs | Observational studies (cross-sectional surveys, cohort studies, designed to identify factors associated with coverage/utilization).  Intervention studies: randomized trials, quasi-experimental study (time-series or before-and-after studies), ecological designs that evaluated outcome evaluation (e.g. ITNs coverage/uptake/effectiveness.  Quantitative, qualitative and mixed-method studies that addressed bed net utilization among MMPs. Also, opinion surveys involving MMPs and stakeholders or the MARC about ITNs.  We, therefore, included studies that addressed knowledge acquisition, net-owning, net-usage in malaria prevention context through public, private, aid agencies or combined delivery systems targeted to the MMPs in MARC. |
| Exclusion criteria | Studies were excluded if: i) they were not reported in English, ii) they did not describe the MSA, iii) they did not target to the MMPs in the MARC zone. We also excluded personals in the arm-forced services and permanent migrants from MMPs due to particular characteristic of their work/situations. mobile populations” (defined as temporary victors, recent travellers, and those who spent the night in the forest [20]. |
