# Supplementary material for: Multisectoral Approach to Support Use of Insecticide-Treated Net for Malaria Prevention Among Mobile and Migrant Populations in Myanmar: A Systematic Review
Source: J Infect Dis. 2020 Oct 29;222(Suppl 8):S717–25. doi: 10.1093/infdis/jiaa335 (PMC7594345; doi:10.1093/infdis/jiaa335)
Supplement: jiaa335_suppl_Supplementary_Table_3 [file jiaa335_suppl_supplementary_table_3.doc]

Supplementary Table 3 Excluded studies and main reason for exclusion

| Study | Reason | Citation |
| --- | --- | --- |
| Win, 2017 | Malaria treatment aspect, not malaria prevention/bed net delivery | Win AYN, Maung TM, Wai KT, et al. Understanding malaria treatment-seeking preferences within the public sector amongst mobile/migrant workers in a malaria elimination scenario: a mixed-methods study. Malar J. 2017;16(1):462. |
| Wangroongsarb,  2011 | Malaria treatment aspect only | Wangroongsarb P, Satimai W, Khamsiriwatchara A, et al. Respondent-driven sampling on the Thailand-Cambodia border. II. Knowledge, perception, practice and treatment-seeking behaviour of migrants in malaria endemic zones. Malar J. 2011;10:117. |
| Zhou, 2016 | Not revealed the setting in the MARC zone | Zhou G, Lo E, Zhong D, et al. Impact of interventions on malaria in internally displaced persons along the China-Myanmar border: 2011-2014. Malar J. 2016 ;15:471. |
| Oo, 2018 | Only a protocol | Oo WH, Cutts JC, Agius PA, et al. Effectiveness of repellent delivered through village health volunteers on malaria incidence in villages in South-East Myanmar: a stepped-wedge cluster-randomised controlled trial protocol. BMC Infect Dis. 2018;18(1):663. |
| Nwe, 2017 | No particular for MMPs | Nwe TQ, Oo T, Wai KT, et al. Malaria profiles and challenges in artemisinin resistance containment in Myanmar Infect Dis Poverty. 2017; 6: 76. |
| Maung, 2018 | No specific data for MMPs | Maung TM, Tripathy JP, Oo T, et al. Household ownership and utilization of insecticide-treated nets under the regional artemisinin resistance initiative in Myanmar. Trop Med Health. 2018; 46:27. |
| Zhang,2016 | Not revealed the setting in the MARC zone | Zhang J, Dong JQ, Li JY, et al. Effectiveness and impact of the cross-border healthcare model as implemented by non-governmental organizations: case study of the malaria control programs by health poverty action on the China-Myanmar border. Infect Dis Poverty. 2016;5:80. |
| Nyunt,2017 | Not assessed treated bed nets | Nyunt MY, Soe MT, Myint HW, et al. Clinical and molecular surveillance of artemisinin resistant falciparum malaria in Myanmar (2009–2013). Malar J. 2017; 16: 333. |
| Wang, 2014 | Not revealed the setting in the MARC zone | Wang R, Zhang J, Zhang O-F. Malaria baseline survey in four special regions of northern Myanmar near China: a cross-sectional study. Malar J. 2014; 13: 302. |
| Mullany, 2010 | Maternal care package | Mullany LC, Lee TJ, Yone L, et al. Impact of community-based maternal health workers on coverage of essential maternal health interventions among internally displaced communities in eastern Burma: the MOM project. PLoS Med. 2010;7:e1000317 |
